# Supplementary material for: Hypercarnivorous teeth and healed injuries to Canis chihliensis from Early Pleistocene Nihewan beds, China, support social hunting for ancestral wolves
Source: PeerJ. 2020 Sep 8;8:e9858. doi: 10.7717/peerj.9858 (PMC7485486; doi:10.7717/peerj.9858)
Supplement: Supplemental Information 1 [file peerj-08-9858-s001.docx]

**Hypercarnivorous teeth and healed injuries to Canis chihliensis from early Pleistocene Nihewan beds, China, support social hunting for ancestral wolves**

Hao-wen Tong^1,2,*^, Xi Chen^3^, Bei Zhang^1,2,4^, Bruce Rothschild^5^, Stuart C. White^6^, Mairin Balisi^7^, and Xiaoming Wang^1,7,*^

^1^ Key Laboratory of Vertebrate Evolution and Human Origins of Chinese Academy of Sciences, Institute of Vertebrate Paleontology and Paleoanthropology, Chinese Academy of Sciences, Beijing, China

^2^ CAS Center for Excellence in Life and Paleoenvironment, Beijing, China

^3^ Nanjing Normal University, Nanjing, China

^4^ University of Chinese Academy of Sciences, Beijing, China

^5^ Department of Vertebrate Paleontology, Carnegie Museum of Natural History, Pittsburgh, Pennsylvania, USA

^6^ School of Dentistry, University of California, Los Angeles, California, USA

^7^ Natural History Museum of Los Angeles County, California, USA.

# Supplementary Information

The following are additional figures cited in the main text.


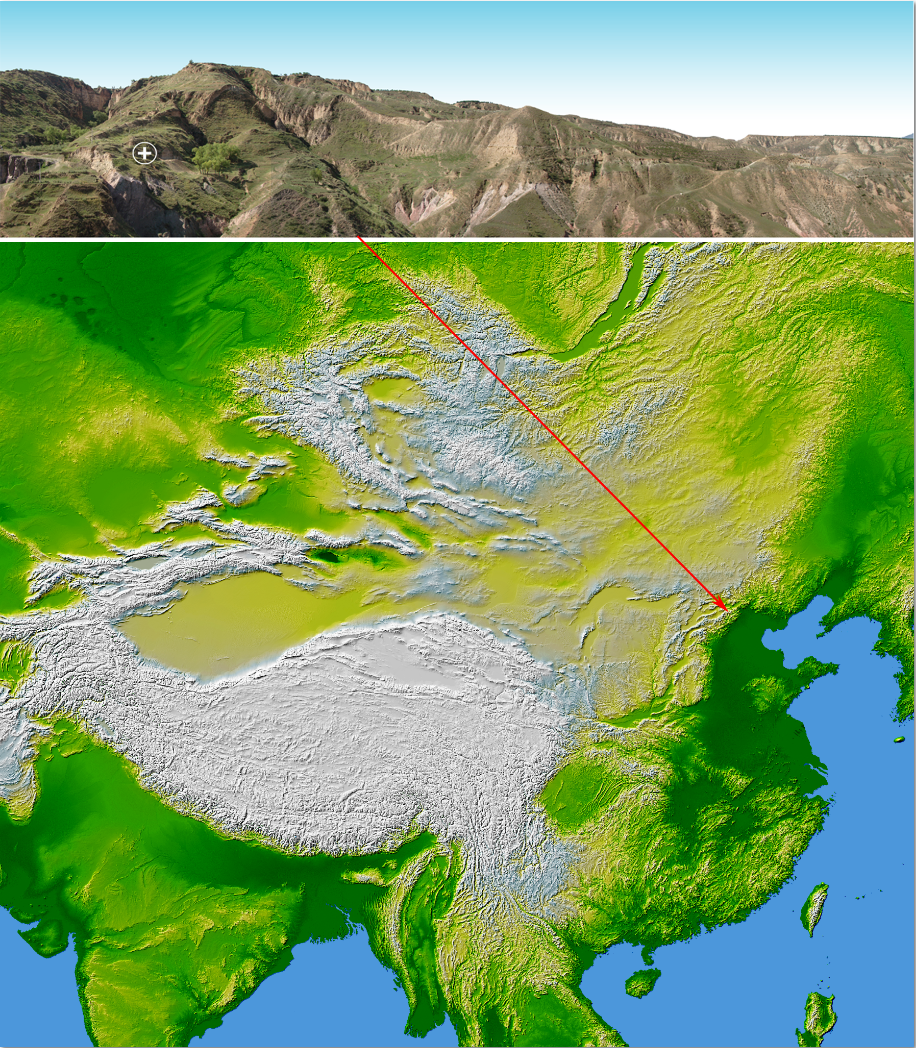


**Figure S1 Photograph of the Shanshenmiaozui Site and map location of the Nihewan Basin.** Topographic image is modified from NASA Earth Observatory (https://earthobservatory.nasa.gov/images/3741/topography-of-the-world).


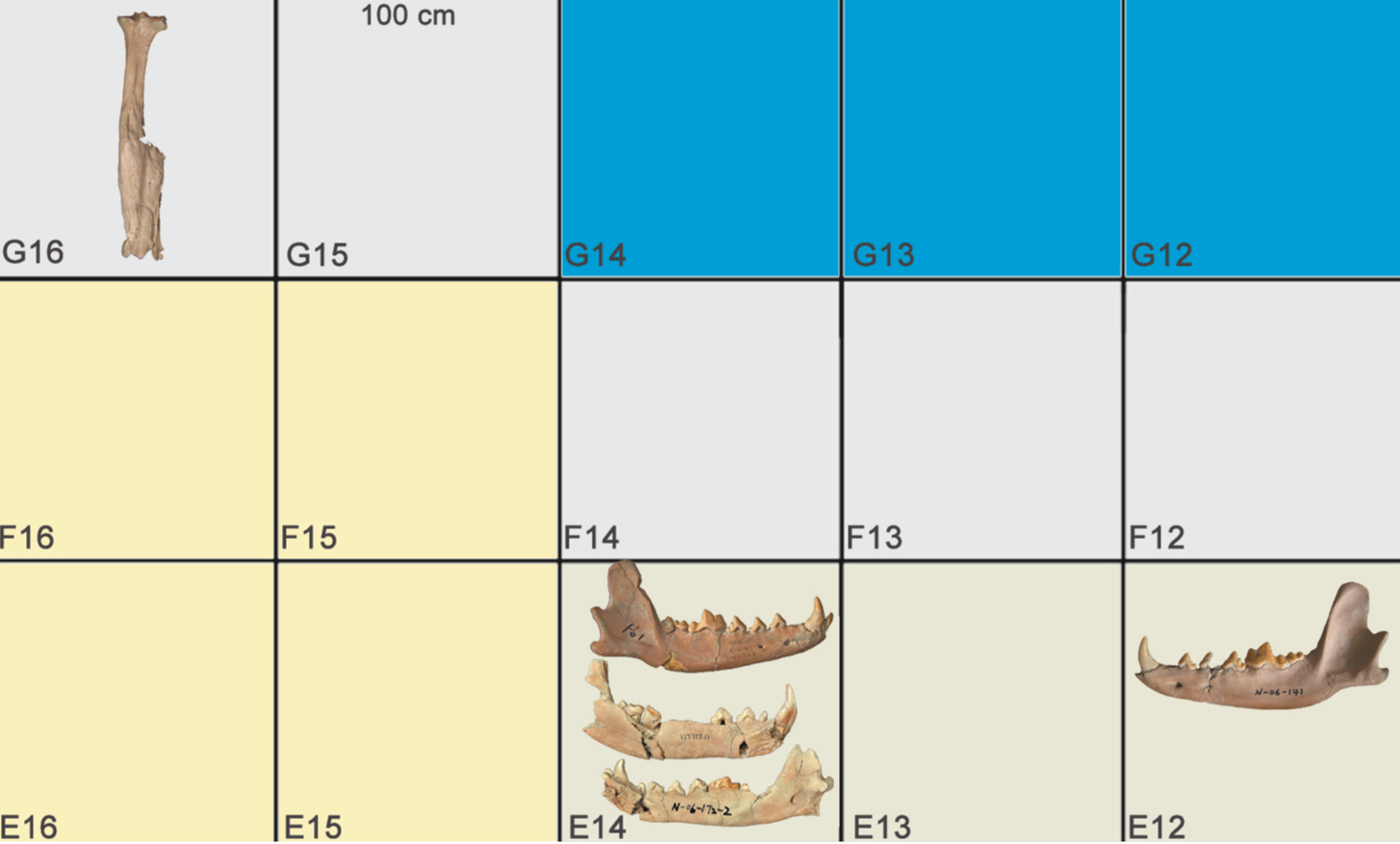


**Figure S2 Excavation grid (1 x 1 m) with representative *Canis* specimens discussed in this paper.**


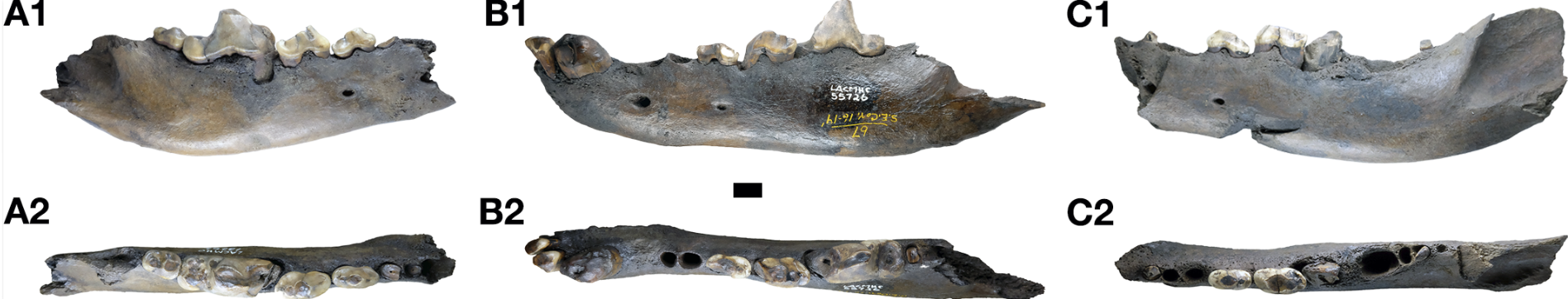


**Figure S3 Examples of dentaries of *Canis dirus* from the late Pleistocene Rancho La Brea asphalt seeps bearing abscesses, alveolar resorption, and tooth fracture in the p4-m1 region similar to those in the pathological *C. chihliensis* dentaries.** A1, B1, and C1 lateral views; A2, B2, and C2 occlusal views. Scale bar = 1 cm. A, LACMHC 13262, right dentary with heavy occlusal wear and alveolar abscess with root drainage on the anterior m1, from Pit 4; B, LACMHC 55726, left dentary with heavy occlusal wear, possible abscess around the anterior alveoli of p4 and m1, and m1 fractured anteriorly, from Pit 61/67; C, LACMHC 55730, left dentary with heavy occlusal wear, fractured m1, and alveolar reaction around the roots, also from Pit 61/67.


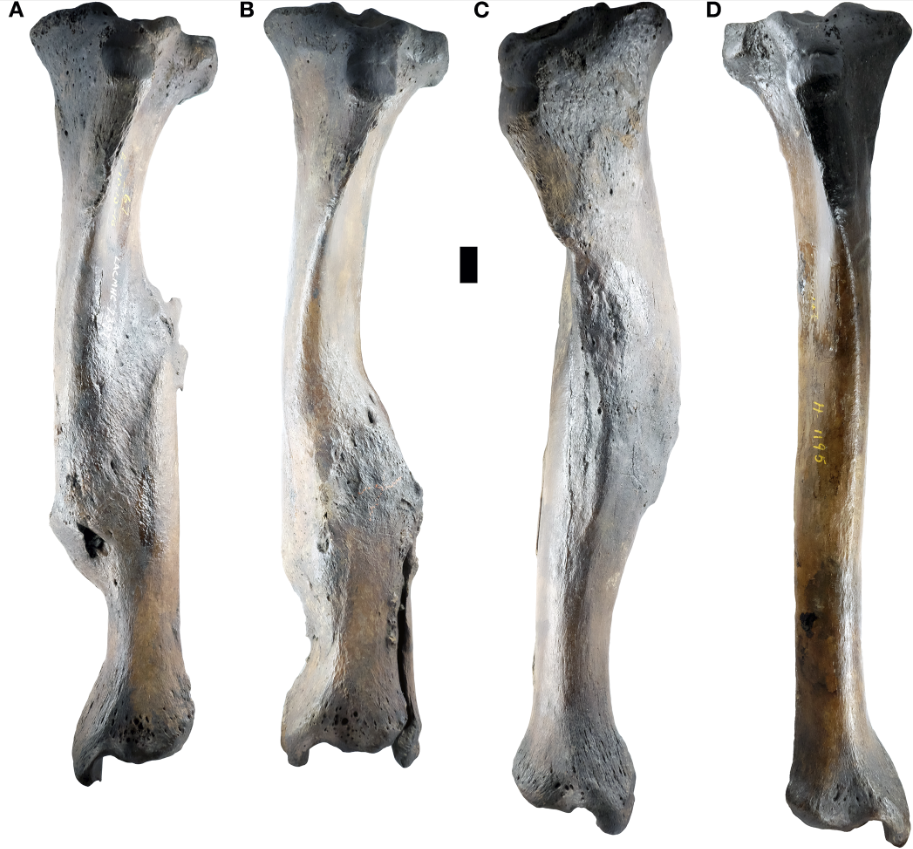


**Figure S4 Examples of tibiae of *Canis dirus* from the late Pleistocene Rancho La Brea asphalt seeps bearing healed oblique fractures of the mid-shaft with foreshortening of the bone, similar to that in the pathological *C. chihliensis* tibia (Figs. 4, 5).** Scale bar = 1 cm. A, LACMHC 7397, left tibia, from Pit 61/67; B, LACMHC 7396, left tibia with distal fibula attached and involved in the oblique fracture, from Pit 61/67; C, LACMHC 7336, right tibia with fused fibula fragment, from Pit 61/67; D, LACMHC H-1195, non-pathologic right tibia for comparison, no deposit data available.
